# Differential reporting of fruit and vegetable intake among youth in a randomized controlled trial of a behavioral nutrition intervention

**DOI:** 10.1186/s12966-019-0774-9

**Published:** 2019-02-01

**Authors:** Namrata Sanjeevi, Leah Lipsky, Aiyi Liu, Tonja Nansel

**Affiliations:** 1 Social and Behavioral Sciences Branch, Eunice Kennedy Shriver National Institute of Child Health and Human Development, Bethesda, MD 20817 USA; 2 Biostatistics & Bioinformatics Branch, Eunice Kennedy Shriver National Institute of Child Health and Human Development, Bethesda, USA

**Keywords:** Differential reporting bias, Carotenoids, Fruit and vegetable intake, Randomized controlled trial, Type 1 diabetes

## Abstract

**Background:**

Nutrition interventions typically rely on self-reported intake that may be susceptible to differential reporting bias due to exposure to the intervention. Such differences may result from increased social desirability, increased attention to eating or improved recall accuracy, and may bias estimates of the intervention effect. This study investigated differential reporting bias of fruit and vegetable intake in youth with type 1 diabetes participating in a randomized controlled trial targeting increased whole plant food intake.

**Methods:**

Participants (treatment *n* = 66, control *n* = 70) completed 3-day food records at baseline, 6-,12-, and 18-months, from which fruit and vegetable intake (servings/day) was calculated. Serum carotenoids were assessed at these visits using a high-performance liquid chromatography-based assay. Linear regression estimated associations of fruit and vegetable intake with serum carotenoids by treatment assignment. Multiplicative interaction terms tested the interaction of treatment assignment with fruit and vegetable intake on serum carotenoids for each visit and within each group over time.

**Results:**

The association of fruit and vegetable intake with serum carotenoids was significantly lower in the control versus intervention group at baseline (β = 0.22 Vs 0.46) and 6-month visits (β = 0.37 Vs 0.54), as evidenced by significant interaction effects. However, the association of fruit and vegetable intake with serum carotenoids did not significantly differ over time for either group.

**Conclusions:**

While the stronger association of fruit and vegetable with carotenoids in the treatment arm suggests greater reporting accuracy, this difference was evident at baseline, and did not change significantly over time in either group. Thus, results indicate greater subject-specific bias in the control arm compared to the treatment, and lack of evidence for reactivity to the intervention by treatment assignment.

**Clinical trial registry number and website:**

NCT00999375

**Electronic supplementary material:**

The online version of this article (10.1186/s12966-019-0774-9) contains supplementary material, which is available to authorized users.

## Background

Self-reported dietary intake obtained from 24-h recalls, food records, and food frequency questionnaires (FFQ) is subject to measurement error attributed to recall bias and social desirability [1]. Such measurement error may increase erroneous inferences in nutrition research, typically biasing estimates towards null. For example, greater social desirability was related to underreporting of caloric intake by women [2], and attenuated the association of body mass index (BMI) with energy intake in African American girls [3]. In randomized controlled trials (RCTs), considered the gold standard for evaluating the efficacy and effectiveness of dietary interventions [4], an additional concern arises due to potential differences in misreporting due to exposure to the intervention [5, 6]. Treatment group participants may increase misreporting of intake in a manner consistent with the target dietary behaviors, or conversely, improve accuracy of self-reports compared to control group by increasing attention to intake. Both scenarios lead to differential reporting error which may bias estimates of the true intervention effect.

Despite concerns about differential reporting error in dietary intervention research [7], few studies have systematically evaluated misreporting bias in RCTs using objective markers of intake (real-time observations of intake or biomarkers), with mixed findings. While most studies indicated greater misreporting of select dietary components in the intervention arm relative to control [8–10], one study reported no differences [11], and another found increased reporting accuracy over time in the intervention group [1]. Additional studies are, therefore, warranted to better understand the extent and nature of reactivity bias and inform strategies to mitigate it. Further, except for one trial [1], differential response bias was explored only at a single follow-up visit. Estimation of differential reporting error across multiple follow-up assessments would inform the timing of reactivity bias in intervention trials. The goal of this study was to examine whether there was differential reporting bias of fruit and vegetable intake at 6, 12 and 18 months follow-up in youth with type 1 diabetes participating in a RCT of a family-based behavioral intervention.

## Methods

### Participants

Parent-youth dyads were recruited for a RCT of a nutrition intervention conducted from August 2010–May 2013 at a tertiary diabetes center in the northeast United States [12]. Eligibility criteria for youth included: age 8.0–16.9 years, diagnosed with type 1 diabetes for at least 1 year, insulin dose of at least 0.5 units/kg/day, glycated hemoglobin (HbA1c) ranging from 6.5 to 10.0% at the most recent clinic visit, use of insulin pump or an insulin regimen of at least 3 injections/day, at least one clinic visit in the previous year, and English communication ability. Youth were excluded if they: used premixed insulin daily, transitioned to insulin pump therapy in the previous 3 months, used real-time continuous glucose monitoring in the previous 3 months, participated in another intervention study in the previous 6 months, or had significant mental illness, gastrointestinal disease such as celiac disease, multiple food allergies, or used medications that interfere significantly with glucose metabolism. Of 622 eligible youth invited to participate, 148 (24%) provided consent and 139 (22%) completed baseline measures. Assessments from one sibling each in 3 sibling pairs were eliminated, resulting in 136 families. Children and adolescents provided assent, while written informed consent was obtained from parents during enrollment. Youth turning 18 years of age during the study also provided written informed consent. The procedures were approved by the institutional review boards of the institutions participating in the study.

### Design

This was a randomized clinical trial with one group receiving a behavioral nutrition intervention (*n* = 66) and the other group serving as the control (*n* = 70) [12]. The nutrition intervention consisted of nine sessions, grounded in behavioral theory, and aimed to increase youth intake of whole plant foods (whole fruits, vegetables, whole grains, legumes, nuts, and seeds). Control group participants had equal number of contacts with research staff, but did not receive any dietary advice besides that given as part of standard diabetes care. During the consent process, participants were informed that families in the intervention group would receive guidance and strategies to improve the quality of their food choices. Participants in both the treatment and control arm completed 3-day diet records and laboratory assessments at baseline, 6, 12, and 18 months.

### Dietary intake

Families completed 3-day youth diet records immediately following each clinic visit (baseline, 6, 12 and 18 months). Families were instructed to report food and beverage intake, including details such as brand/restaurant names and fat composition of foods. Families received a sample diet record including instructions on reporting portion size, mode of food preparation, and addition of fat/ condiments. Further, measuring cups and spoons were given to enable portion size estimation. Research staff reviewed the records for completeness and contacted families to clarify any necessary missing information. A registered dietitian administered two non-consecutive 24-h dietary recalls if diet records were not completed (1.7% of assessments). The Nutrition Data System for Research (NDSR 2012; Nutrition Coordinating Center, University of Minnesota) was used to provide estimates of fruit and vegetable servings and micronutrient intake. Fruit and vegetable servings were summed to represent total intake. Daily carotenoid intake was calculated from the sum of α-carotene, β-carotene, lutein/zeaxanthin, lycopene, and β-cryptoxanthin.

### Serum carotenoids

Blood samples collected at baseline, 6, 12 and 18 months were stored at room temperature for 20–30 min, followed by centrifugation for 15 mins at ~ 3000 RPM at 4 °C. Samples were then aliquoted and frozen at − 80 °C for later assay. A high-performance liquid chromatography-based assay was used to assess serum concentrations of α-carotene, β-carotene, lycopene and β-cryptoxanthin and lutein plus zeaxanthin. Day-to-day coefficient of variation for the carotenoids was approximately 7%. Concentration of lutein/zeaxanthin was converted from μmol/L to μg/mL, and summed with other carotenoids (expressed as μg/mL) to represent total serum carotenoids. Serum carotenoids reflect intake over the previous one to two weeks [13].

### Clinical and demographic data

Age, sex, height and weight were abstracted from medical records, and multivitamin use was obtained from self-reports. BMI (kg/m^2^), calculated from measured height and weight, was transformed to z-scores. Enzyme linked immunosorbent assay was used to measure serum low-density lipoprotein cholesterol (LDL-C) and high-density lipoprotein cholesterol (HDL-C). An assay standardized to the Diabetes Control and Complications Trial (reference range, 4–6%, [20–42 mmol/mol]) was used to assess HbA1c. Initially, Tosoh (Tosoh Medics) was used to conduct HbA1c assays followed by Roche Cobas Integra. Laboratory analysis of results from the two instruments on identical samples indicated no clinically significant bias.

### Analysis

Baseline participant demographic and clinical characteristics were summarized using descriptive statistics, and baseline differences between treatment and control arms were examined using *t*- and chi-square tests for continuous and categorical variables, respectively. Multiple imputation using the fully conditional specification method [14] was used to deal with missing data for the independent variables, dependent variables and covariates. The proportion of participants (%) who completed the 3-day diet records at baseline, 6, 12 and 18 months was 100, 89.7, 87.5 and 89.0%, respectively. Blood samples for carotenoid assessment were drawn from 98.5, 91.2, 89.7 and 89.0% of participants at baseline, 6, 12 and 18 months, respectively. The number of multiple imputations (*n* = 13) was set to be similar to the maximum percentage of missing data [15, 16] based on all visits. The minimum value that can be imputed was set based on the observed minimum from the observed data. Linear regression analysis was used to estimate the association of fruit and vegetable intake with serum carotenoids in treatment and control groups at baseline, 6, 12 and 18 months follow up. Analyses adjusted for age, sex, HDL-C, LDL-C, BMI z-score, HbA1c and multivitamin use. The slope of this regression analysis indicated the correlation of diet-serum carotenoids. However, the slope would remain unchanged in case of consistent over-reporting of fruit and vegetable intake by intervention group participants. In contrast, constant systematic error, as indicated by y-intercept deviation from 0, can capture consistent misreporting of intake. Thus, the difference in y-intercept between treatment and control was used to explore constant error at every time point [17].

Consider the regression equation for the treatment as: mean serum carotenoids = β_0_ + β_1_ (fruit and vegetable intake), and that for control as: mean serum carotenoids = β_2_ + β_3_ (fruit and vegetable intake).

It is assumed that a fruit and vegetable intake of 0 servings/day must indicate lower serum carotenoids, and thus a smaller y-intercept. Thus, greater y-intercepts in the treatment compared to control group would indicate greater reporting error in the treatment group. The magnitude and *p*-value of the difference in intercepts between treatment and control (β_0_ - β_2_) was obtained by regressing treatment assignment and fruit and vegetable intake over serum carotenoids for the entire sample. Thus, the relationship is represented by the equation.

Mean serum carotenoids = β_4_ + β_5_ (treatment assignment) + β_6_ (fruit and vegetable intake) where β_5_ indicates the difference in intercepts. This analysis was not adjusted for covariates (age, sex, HDL-C, LDL-C, BMI z-score, HbA1c and multivitamin use) to meaningfully interpret the difference in constant systematic error between the treatment and control arms. A similar analysis was used to explore difference in constant systematic error over time within the intervention and control groups.

Multiplicative interaction terms tested whether treatment assignment moderated the association of fruit and vegetable intake (centered at the mean) with serum carotenoids for each visit. Similarly, multiplicative interaction terms estimated whether timeline of visit moderated the association of fruit and vegetable intake (centered at the mean) with serum carotenoids for the intervention and control group. A three-way interaction between fruit and vegetable intake (centered at the mean), treatment assignment and the timeline of visit on serum carotenoids also was conducted to determine whether the moderating effect of treatment assignment on relationship of fruit and vegetable intake with serum carotenoids differed based on visit timeline. The interaction analyses controlled for age, sex, HDL-C, LDL-C, BMI z-score, HbA1c and multivitamin use. Linear mixed effects models estimated association of time-varying fruit and vegetable intake with time-varying serum carotenoid concentrations. Models included a random intercept to represent the subject-specific baseline variation in the outcome, and were adjusted for age, treatment assignment, sex, HDL-C, LDL-C, BMI z-score, HbA1c and multivitamin use. Multiplicative interaction terms of intake with treatment assignment were used to estimate whether treatment assignment moderated the relationship of intake with serum carotenoids in a longitudinal model.

The above-mentioned analyses to estimate diet-serum carotenoids correlation, constant systematic error, and moderating effect of treatment assignment and visit timeline were also conducted using carotenoid intake as the independent variable. SPSS version 21 was used for all analyses.

## Results

### Participant flow chart and baseline characteristics

**Fig. 1:**
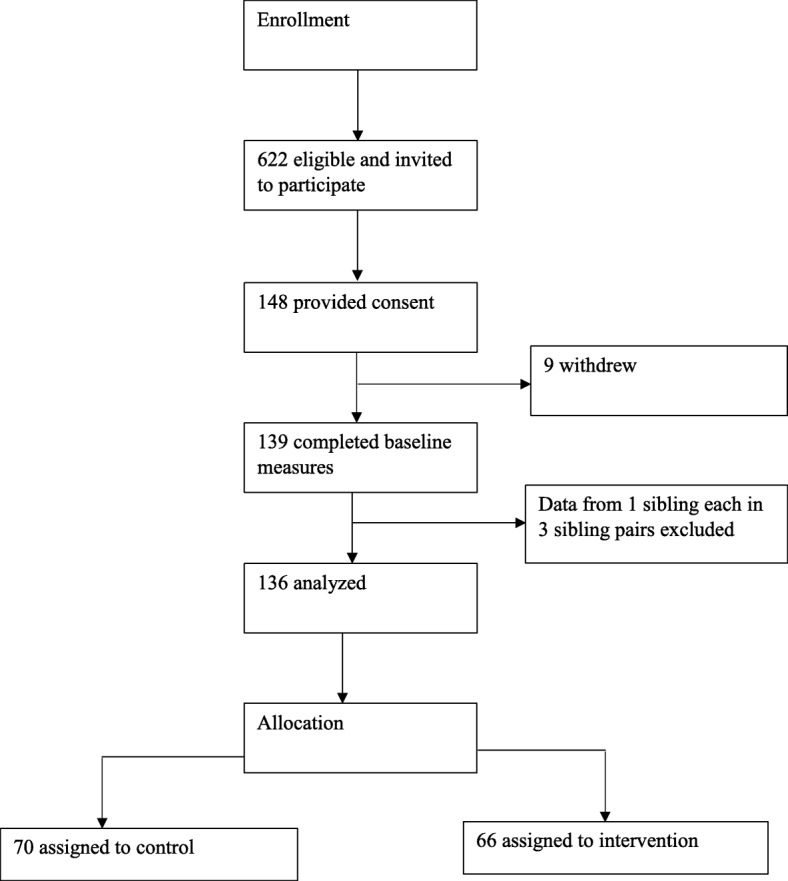
Participant flow chart of a randomized controlled trial of a behavioral nutrition intervention for youth with type 1 diabetes

Figure 1 indicates participant flow from recruitment to treatment assignment. Baseline characteristics did not significantly differ between the treatment and control groups (Table 1).

**Table 1:** Baseline sample characteristics^a^ of youth with type 1 diabetes participating in a behavioral nutrition intervention

	Overall (*n* = 136)	Intervention (*n* = 66)	Control (*n* = 70)	*p*
Age, years	12.7 ± 2.6	12.5 ± 2.7	13.0 ± 2.5	0.27
Body mass index, kg/m2	21.3 ± 4.2	21.0 ± 4.1	21.6 ± 4.3	0.37
HbA1c^b^, (%)	8.1 ± 1.0	8.1 ± 1.1	8.1 ± 1.0	0.95
Duration of diabetes, years	6.0 ± 3.1	5.6 ± 2.5	6.4 ± 3.6	0.15
Youth race/ethnicity				
Non-Hispanic white	123 (90.4)	58 (87.9)	65 (92.9)	0.17
Non-Hispanic black	5 (3.7)	2 (3.0)	3 (4.3)	
Hispanic	7 (5.2)	6 (9.1)	1 (1.4)	
American Indian/Alaska Native	1 (0.7)	0 (0)	1 (1.4)	
Use of multivitamin supplement				
Yes	51 (37.5)	19 (28.8)	32 (45.7)	0.05
No	85 (62.5)	47 (71.2)	38 (54.3)	
Low density lipoprotein-cholesterol	86.35 ± 23.96	85.66 ± 19.68	87.00 ± 27.53	0.75
High density lipoprotein-cholesterol	56.55 ± 1.17	56.52 ± 13.97	56.58 ± 13.30	0.98
Fruit and vegetable intake, servings/day	1.69 ± 0.09	1.77 ± 0.13	1.63 ± 0.12	0.36
Serum carotenoids, μg/ml	1.44 ± 0.68	1.45 ± 0.10	1.43 ± 0.07	0.85

^a^Values are mean ± standard deviation or *n* (%)

^b^HbA1c, Glycated hemoglobin

### Moderating effect of treatment assignment and visit timeline on relationship of fruit and vegetable intake with serum carotenoids

**Fig. 2:**
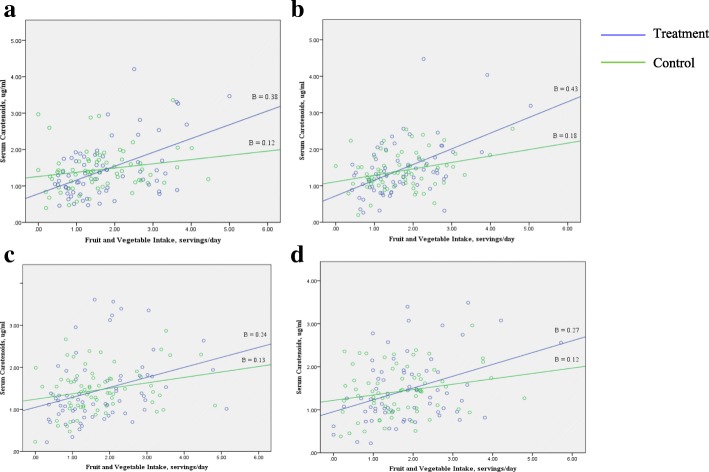
Moderation of fruit and vegetable intake and serum carotenoids relationship by treatment assignment at baseline (**a**), 6 (**b**), 12 (**c**) and 18-month (**d**) follow-up visit

Table 2 indicates the standardized coefficient estimates for the association between fruit and vegetable intake and serum carotenoids based on treatment assignment for each visit from baseline. The adjusted coefficient estimate of the intervention group was greater than that of the control for all visits except for the 12-month follow up. There was a significant interaction between treatment assignment and fruit and vegetable intake for the baseline visit (β = − 0.52, *p* = 0.04) and 6-month follow up (β = − 0.52, *p* = 0.01). The regression coefficient was larger for the intervention group compared to control at baseline and 6 months (Fig. 2a & 2b), whereas this difference was not found at 12 and 18 months (Fig. 2c & 2d). There was no interaction of visit timeline with fruit and vegetable intake on serum carotenoids within the treatment or control arm. The 3-way interaction between fruit and vegetable intake, treatment assignment and visit timeline was not significant. Further, treatment assignment did not moderate the relationship of intake with serum carotenoids in the longitudinal analysis.

**Table 2:** Association of reported fruit and vegetable intake with serum carotenoids^a^ for intervention and control groups at baseline and follow up

Visit timeline	Intervention		Control	
	β^b^	*p*	β^b^	*P*
Baseline	0.45	< 0.001	0.23	0.07
6 months follow-up	0.54	< 0.001	0.38	< 0.001
12 months follow-up	0.29	0.02	0.26	0.04
18 months follow-up	0.28	0.009	0.29	0.03

^a^Estimated by regressing fruit and vegetable intake on serum carotenoids

^b^Adjusted for age, sex, high-density lipoprotein cholesterol, low-density lipoprotein cholesterol, BMI z-score, glycated hemoglobin and multivitamin use

### Moderating effect of treatment assignment and visit timeline on relationship of carotenoid with serum carotenoids

Additional file 1: Table S1 indicates the standardized coefficient estimates for the association of carotenoid intake with serum carotenoids based on treatment assignment for each visit from baseline. The adjusted coefficient estimate of the intervention group was greater than that of the control at baseline and 12-month follow up, and was not significant at the 18-month follow up. There was a significant interaction between treatment assignment and carotenoid intake for the 12-month follow up (β = − 0.66, *p* = 0.03). There was no interaction of visit timeline with carotenoid intake on serum carotenoids within the treatment or control arm. The 3-way interaction between carotenoid intake, treatment assignment and visit timeline was significant (β = 0.17, *p* < 0.001); however, treatment assignment did not moderate the relationship of intake with serum carotenoids in the longitudinal analysis.

### Constant systematic error in reporting of fruit and vegetable intake

Table 3 indicates lack of significant differences in constant systematic error between the intervention and control groups at baseline, 6, 12 and 18-months follow-up. Further, there were no significant differences in constant error over time in either the intervention or control group (Table 4).

**Table 3:** Difference in constant systematic error in reporting of fruit and vegetable intake between intervention and control groups

Visit timeline	β^a^	*p*
Baseline	0.02	0.78
6 months follow-up	−0.03	0.70
12 months follow-up	0.004	0.96
18 months follow-up	−0.001	0.99

^a^Estimated by regressing treatment assignment and fruit and vegetable intake on serum carotenoids, where the slope of treatment assignment indicates difference in constant systematic error between intervention and control

**Table 4:** Difference in constant systematic error in reporting of fruit and vegetable intake between baseline and each follow-up visit

Visit timeline	Intervention		Control	
	β^a^	*p*	β^a^	*p*
Baseline, 6 months follow-up	0.03	0.73	−0.02	0.83
Baseline, 12 months follow-up	−0.03	0.68	0.04	0.65
Baseline, 18 months follow-up	−0.06	0.50	−0.001	0.99

^a^Estimated by regressing visit timeline and fruit and vegetable intake on serum carotenoids, where the slope of visit timeline indicates difference in constant systematic error between baseline and follow-up visit

### Constant systematic error in reporting of carotenoid intake

Additional file 2: Table S2 and Additional file 3: Table S3 indicate lack of significant difference in constant systematic error between the intervention and control groups, and over time, respectively.

## Discussion

Differential response bias in nutrition intervention research may arise when the treatment group responds to the intervention by either improving or decreasing self-report accuracy of dietary intake. In the current study, differential response bias would be evidenced by a) a difference in the magnitude of association of intake with serum carotenoids or in constant systematic error within the intervention group from baseline to follow-up, and/or b) a difference in the magnitude of association of intake with serum carotenoids or in constant systematic error between the intervention and control group that arises after baseline. However, neither of these were observed. The association of fruit and vegetable intake with serum carotenoids was greater in the treatment group compared to control; however, this difference was observed at baseline, and the association did not vary significantly over time in the treatment and control arms. Similarly, the association of carotenoid intake with serum carotenoids was greater in the treatment group compared to control at baseline, and did not vary by visit timeline in each arm. However, the association of carotenoid intake with serum carotenoids was not significant at the 18-month follow up. This could be attributed to possible misreporting of carotenoid sources which were not intervention targets. Further, the difference in constant systematic error of self-reported fruit and vegetable and carotenoid intake between the intervention and control was not significant, and did not vary over time. Collectively, the results are not consistent with differential response bias in reporting of dietary intake due to exposure to the intervention.

Substantial discrepancies have been observed across the literature on differential dietary reporting in interventions. While there was little evidence of differential response bias in the current study, a study in adult breast cancer survivors using serum carotenoids to examine reporting bias indicated improved validity, but increased systematic error of self-reported fruit and vegetable intake over time in the intervention group [1]. In contrast, other studies have shown greater misreporting of macronutrient (energy, fat [8] and protein [10]) and micronutrient (sodium and potassium [9]) intakes by the intervention arm of RCTs. However, these nutrients were not intervention targets in the current study. Increased attention to diet already present in families of youth with type 1 diabetes (due to the central role of diet in disease management) may have mitigated the potential for improved accuracy among the intervention group. However, it is less likely that diabetes management would diminish misreporting of intake behaviors targeted by the intervention. Standard dietary advice given to these patients differs substantially in content and approach than this intervention, which specifically focused on intake of whole plant foods via nutrition education, goal setting, barrier identification, and motivational interviewing. Diet-serum relationship at 12 and 18 months are comparable to another study reporting no differences in self-reported and observed fruit and vegetable intake between the treatment and control arms of a post-intervention study in fourth-grade children [11]. Given the heterogeneous results between studies, additional research is needed to further explore differential reporting bias in RCTs.

Differential reporting bias due to intervention exposure is a potential source of measurement error unique to experimental research [1, 9, 10]. However, irrespective of intervention exposure, measurement error due to person-specific bias may also occur in self-reports. In this study, greater diet-serum carotenoid correlations in the intervention compared to control was observed at baseline and 6 months follow-up. Since the difference was observed at baseline, this cannot be interpreted as evidence of reactivity to the intervention that had not yet transpired. This finding also cannot be attributed to participant demographics associated with greater reporting error (for example, age and BMI) [10] as these characteristics did not differ between the two groups. However, this result corroborates previous research on subject-specific error in self-reported dietary intake [18, 19].

Results of this study must be interpreted in light of its limitations. Since there are no recovery biomarkers for fruit and vegetable intake, the absolute degree of bias in self-reported intake cannot be inferred. However, the analyses controlled for several covariates that are known to impact carotenoid bioavailability [20]. Another limitation is that the blood draw for serum carotenoid measurement preceded dietary record completion. However, since no intervention components were provided in this interval and for one month prior to blood sampling, dietary intake reflected by serum carotenoids is not expected to significantly differ from that following specimen collection. The study included a small sample of youth with type 1 diabetes, mostly of white race, thereby limiting generalizability of results.

## Conclusions

This study explored sources of error in dietary self-reports that could limit interpretation of effect size in RCTs. Greater subject-specific bias in self-reported fruit and vegetable intake was evident in the control arm compared to the treatment, thus implying the need to use biomarkers whenever possible to supplement self-reports in intervention studies. Further, intervention effect estimate must be evaluated given this difference in dietary reporting between the control and treatment arms. However, this study does not provide evidence for reactivity bias in self-reports within the treatment arm. Thus, it is reasonable to conclude that differential reporting error could not have biased intervention effects in this study.

## Additional files

Additional file 1: Association of reported carotenoid intake with serum carotenoids for intervention and control groups at baseline and follow up. (DOCX 14 kb)

Additional file 2: Difference in constant systematic error in reporting of carotenoid intake between intervention and control groups. (DOCX 13 kb)

Additional file 3: Difference in constant systematic error in reporting of carotenoid intake between baseline and each follow-up visit. (DOCX 14 kb)

## Abbreviation

BMI: Body mass index
ELISA: Enzyme linked immunosorbent assay
FFQ: Food frequency questionnaire
HbA1c: Glycated hemoglobin
HDL-C: High-density lipoprotein cholesterol
LDL-C: Low-density lipoprotein cholesterol
RCT: Randomized controlled trial
