# Supplementary material for: Differential reporting of fruit and vegetable intake among youth in a randomized controlled trial of a behavioral nutrition intervention
Source: Int J Behav Nutr Phys Act. 2019 Feb 1;16:15. doi: 10.1186/s12966-019-0774-9 (PMC6359852; doi:10.1186/s12966-019-0774-9)
Supplement: Supplementary file 1 — Association of reported carotenoid intake with serum carotenoids for intervention and control groups at baseline and follow up. (DOCX 14 kb) [file 12966_2019_774_MOESM1_ESM.docx]

Additional file 1: Table S1. Association of reported carotenoid intake with serum carotenoids^a^ for intervention and control groups at baseline and follow up

| Visit timeline | Intervention | | Control | |
| --- | --- | --- | --- | --- |
|  | β^b^ | p | β^b^ | P |
| Baseline | 0.33 | 0.007 | 0.22 | 0.09 |
| 6 months follow-up | 0.31 | 0.006 | 0.51 | <0.001 |
| 12 months follow-up | 0.25 | 0.04 | 0.08 | 0.56 |
| 18 months follow-up | 0.07 | 0.55 | 0.28 | 0.02 |

^a^Estimated by regressing carotenoid intake on serum carotenoids

^b^Adjusted for age, sex, high-density lipoprotein cholesterol, low-density lipoprotein cholesterol, BMI z-score, glycated hemoglobin and multivitamin use
