# Supplementary material for: Differential reporting of fruit and vegetable intake among youth in a randomized controlled trial of a behavioral nutrition intervention
Source: Int J Behav Nutr Phys Act. 2019 Feb 1;16:15. doi: 10.1186/s12966-019-0774-9 (PMC6359852; doi:10.1186/s12966-019-0774-9)
Supplement: Supplementary file 2 — Difference in constant systematic error in reporting of carotenoid intake between intervention and control groups. (DOCX 13 kb) [file 12966_2019_774_MOESM2_ESM.docx]

Additional file 2: Table S2. Difference in constant systematic error in reporting of carotenoid intake between intervention and control groups

| Visit timeline | β^a^ | p |
| --- | --- | --- |
| Baseline | 0.01 | 0.87 |
| 6 months follow-up | -0.02 | 0.80 |
| 12 months follow-up | -0.02 | 0.82 |
| 18 months follow-up | -0.04 | 0.62 |

^a^Estimated by regressing treatment assignment and carotenoid intake on serum carotenoids, where the slope of treatment assignment indicates difference in constant systematic error between intervention and control
