# Supplementary material for: Differential reporting of fruit and vegetable intake among youth in a randomized controlled trial of a behavioral nutrition intervention
Source: Int J Behav Nutr Phys Act. 2019 Feb 1;16:15. doi: 10.1186/s12966-019-0774-9 (PMC6359852; doi:10.1186/s12966-019-0774-9)
Supplement: Supplementary file 3 — Difference in constant systematic error in reporting of carotenoid intake between baseline and each follow-up visit. (DOCX 14 kb) [file 12966_2019_774_MOESM3_ESM.docx]

Additional file 3: Table S3. Difference in constant systematic error in reporting of carotenoid intake between baseline and each follow-up visit

| Visit timeline | Intervention | | Control | |
| --- | --- | --- | --- | --- |
|  | β^a^ | p | β^a^ | p |
| Baseline, 6 months follow-up | 0.04 | 0.62 | -0.004 | 0.96 |
| Baseline, 12 months follow-up | -0.02 | 0.81 | 0.04 | 0.65 |
| Baseline, 18 months follow-up | -0.03 | 0.79 | -0.04 | 0.64 |

^a^Estimated by regressing visit timeline and carotenoid intake on serum carotenoids, where the slope of visit timeline indicates difference in constant systematic error between baseline and follow-up visit
